# Self‐configuring nnU‐Net for automatic delineation of the organs at risk and target in high‐dose rate cervical brachytherapy, a low/middle‐income country's experience

**DOI:** 10.1002/acm2.13988

**Published:** 2023-04-12

**Authors:** Didier Duprez, Christoph Trauernicht, Hannah Simonds, O'Brian Williams

**Affiliations:** ^1^ Division of Medical Physics Stellenbosch University Tygerberg Academic Hospital Cape Town South Africa; ^2^ Department of Oncology University Hospitals Plymouth NHS trust Plymouth UK; ^3^ Division of Radiation Oncology Stellenbosch University Tygerberg Academic Hospital Cape Town South Africa

**Keywords:** cervical cancer, deep learning, high‐dose rate brachytherapy, segmentation

## Abstract

**Background:**

The high‐dose rate (HDR) brachytherapy treatment planning workflow for cervical cancer is a labor‐intensive, time‐consuming, and expertise‐driven process. These issues are amplified in low/middle‐income countries with large deficits in experienced healthcare professionals. Automation has the ability to substantially reduce bottlenecks in the planning process but often require a high level of expertise to develop.

**Purpose:**

To implement the out of the box self‐configuring nnU‐Net package for the auto‐segmentation of the organs at risk (OARs) and high‐risk CTV (HR CTV) for Ring‐Tandem (R‐T) HDR cervical brachytherapy treatment planning.

**Methods:**

The computed tomography (CT) scans of 100 previously treated patients were used to train and test three different nnU‐Net configurations (2D, 3DFR, and 3DCasc). The performance of the models was evaluated by calculating the Sørensen‐dice similarity coefficient, Hausdorff distance (HD), 95^th^ percentile Hausdorff distance, mean surface distance (MSD), and precision score for 20 test patients. The dosimetric accuracy between the manual and predicted contours was assessed by looking at the various dose volume histogram (DVH) parameters and volume differences. Three different radiation oncologists (ROs) scored the predicted bladder, rectum, and HR CTV contours generated by the best performing model. The manual contouring, prediction, and editing times were recorded.

**Results:**

The mean DSC, HD, HD95, MSD and precision scores for our best performing model (3DFR) were 0.92/7.5 mm/3.0 mm/ 0.8 mm/0.91 for the bladder, 0.84/13.8 mm/5.3 mm/1.4 mm/0.84 for the rectum, and 0.81/8.5 mm/6.0 mm/2.2 mm/0.80 for the HR CTV. Mean dose differences (D_2cc/90%_) and volume differences were 0.08 Gy/1.3 cm^3^ for the bladder, 0.02 Gy/0.7 cm^3^ for the rectum, and 0.33 Gy/1.5 cm^3^ for the HR CTV. On average, 65% of the generated contours were clinically acceptable, 33% requiring minor edits, 2% required major edits, and no contours were rejected. Average manual contouring time was 14.0 min, while the average prediction and editing times were 1.6 and 2.1 min, respectively.

**Conclusion:**

Our best performing model (3DFR) provided fast accurate auto generated OARs and HR CTV contours with a large clinical acceptance rate.

## 1 INTRODUCTION

Cervical cancer is an ever‐growing burden in Africa with the 2017 National Centre for Communicable Diseases (NCID) registry recording approximately 6600 new cervical cancer cases in South Africa alone. This accounted for 15.85% of all new cancer cases among women.^1^ In 2018, the World Health Organization (WHO) registered 13 004 new cervical cancer cases, accounting for 12.1% of all cancer cases recorded for men and women combined.^2^ These numbers are expected to only increase over the coming years. It is therefore crucial to ensure that patients gain access to the latest treatment techniques and for health professionals to continuously strive to improve all aspects of the radiotherapy treatment process.

Image guided High Dose Rate (IG‐HDR) intracavitary brachytherapy is an irreplaceable curative treatment modality for locally advanced cervical cancer. Often provided as a primary or boost treatment option, HDR brachytherapy provides steep dose gradients that offer excellent local control rates while reducing a number of normal tissue complications.^3^, ^4^, ^5^, ^6^ Several studies have shown an increase in survival rates and a decrease in recurrence rates when brachytherapy is used as part of a patient's treatment regimen.^7^, ^8^, ^9^, ^10^ Although IG‐HDR brachytherapy comes with great advantages, the treatment planning workflow as a whole is a labor‐intensive process that includes a number of manual, time‐consuming steps or processes and involves input from a range of professionals; Radiation Oncologists (ROs), Radiotherapists (RTs), and Medical Physicists (MPs). While MRI is the gold standard, CT‐based IG brachytherapy is more cost‐effective in a resource‐constrained environment with limited, or no access to MRI.

Organ at risk (OAR) and target delineation is an extremely important aspect of IG brachytherapy; however, this step makes up a significant portion of the whole treatment planning time and depends largely on the expertise of the RO. Several studies have highlighted issues due to inter‐ and intra‐observer variability in organ delineation with Hellebust et al. and Saarnak et al. reporting inter‐observer variabilities between 5−8% and 10−11%, respectively.^11^, ^12^, ^13^, ^14^, ^15^ Not only is inter‐ and intra‐observer variability a growing concern, low‐ and middle‐income countries, such as those in Africa, face a variety of challenges in an attempt to meet the demands for high‐quality cancer treatment. These include and are not limited to equipment, maintenance, high workloads as well as huge deficits in experienced ROs, MPs, and RTs.^16^, ^17^ A recent investigation into the current state of cancer in sub‐Saharan Africa (SSA), published in the Lancet Oncology journal, found that the shortages of radiotherapy professionals is one of the most crucial barriers hindering access to cancer services in SSA, with an estimated 211% increase in workforce required to provide equitable access to radiotherapy.^19^ Computer‐aided automation has the ability to mitigate the bottlenecks experienced throughout the radiotherapy process and to alleviate some of the workload on the ROs, MPs, and RTs. As a result, there has been a growing demand internationally to automate as much of the radiotherapy treatment planning workflow as possible.

Over the past few years, several techniques have been proposed to automate the delineation of OARs and targets in radiotherapy. A commonly used approach is Atlas Based (AB) methods, which involve deformable image registration, where segmentations from the reference image or atlas are transformed onto the new or test image.^19^, ^20^, ^21^, ^22^ AB methods suffer from lack of certainty in deformable registration and provide sub‐optimal segmentations when dealing with atypical patients, organ motion, and varying levels of bladder and rectum filling.^23^, ^24^, ^25^ Due to the inconsistencies in AB methods, there has been a worldwide shift toward deep learning (DL) automated segmentation techniques, providing an increase in accuracy, reproducibility, and robustness.^26^, ^27^, ^28^, ^29^

The automated delineation of OARs and targets using DL networks in external beam (EB) radiotherapy has been successfully applied to cases such as head and neck,^31^ brain metastases,^32^ breast,^28^ rectum,^33^ pelvis,^33^, ^34^ and cervix.^35^, ^36^ DL has also been effectively applied in various areas of brachytherapy from applicator reconstruction, dose calculation, treatment planning as well as organ delineation.^38^ In IG‐HDR cervical brachytherapy, work has been done on automating the reconstruction of applicators using DL,^38^, ^39^ with a few studies looking at the automatic segmentation of the OARs and targets.^39^, ^40^, ^41^, ^42^, ^43^

These studies however do not look at the clinical acceptability of the generated contours and involve complex deep learning models that require a high level of expertise to reproduce or apply in one's own clinical department.^45^ The purpose of this study is to train and implement the self‐configuring No New U‐Net (nnU‐Net), developed by Isensee et al., for the task of automatically delineating the OARs and HR‐CTV in IG‐HDR cervical brachytherapy.^45^, ^46^ To the best of our knowledge, this is the first CT‐based study applying the novel nnU‐Net to cervical brachytherapy segmentation with ring and tandem applicators. This addition is important, as low/middle‐income departments experience severe bottlenecks in their planning process and may not have the required expertise to design and implement their own machine learning models, a task that nnU‐Net was designed to tackle.

## 2 MATERIALS AND METHODS

### 2.1 Clinical dataset

CT images from 100 locally advanced cervical cancer patients were included in the study. All patients were initially treated with external beam radiotherapy (50 Gy/25 fractions or 46 Gy/23 fractions), followed by HDR brachytherapy (25 Gy/5 fractions or 24 Gy/4 fractions) using the ring and tandem applicators. All patients were scanned on a Phillips Brilliance Big Bore 16 Slice CT scanner. Images were reconstructed with a 512 × 512 matrix size and 2 or 3 mm slice thickness. All OARs and HR‐CTVs were subsequently contoured and approved by a clinical oncologist, based on the IBS‐GEC ESTRO‐ABS recommendations.^48^

To maintain patient anonymity, all patient data was completely anonymized. The pixel data from each patient's CT was extracted from the DICOM files and converted into a three‐dimensional (3D) NIFTI file format. The contour information was also extracted from the DICOM structure file and used to create 3D multi‐class segmentation masks of the OARs and HR‐CTVs and saved in NIFTI file format. The 100 patients included in the study were then split into 80 patients for training/validation and 20 patients for testing. The 80 patients were then further split into 64 for training and 16 for validation.

### 2.2 nnU‐Net

Deep learning‐based segmentation models tend to be extremely task specific, with any slight adjustment in architecture, training parameters or data, leading to significant drops in performance. Therefore, one often requires a high level of expertise to design a robust network architecture, optimize the data augmentation, determine the appropriate pre‐processing or post‐processing, and select the ideal training parameters to obtain a model best suited for the segmentation task at hand.^45^ This process is especially cumbersome when dealing with 3D medical images, where image size, imaging modality, voxel size, and class imbalance vary substantially resulting in poor transfer of a configuration from one dataset to another.

As a solution, Isensee et al. developed a self‐configuring method for deep learning‐based image segmentation specific to bio‐medical imaging. The result is a completely automated deep learning segmentation pipeline, allowing any user with minimal programming knowledge the ability to set up and train their very own segmentation models.^45^, ^46^ At its heart, nnU‐Net is based on the U‐Net architecture, developed by the computer science department of the University of Freiburg for biomedical image segmentation.^49^ Isensee et al. have found that a well‐trained U‐Net has the potential to beat or at the very least match any task specific segmentation model, leading them to develop their segmentation pipeline known as No New U‐Net or nnU‐Net. This self‐configuring segmentation pipeline was proven to outperform extremely task specific model architectures in several international biomedical auto‐segmentation competitions on 23 different publicly available datasets. These 23 different datasets include anatomical sites from brain, head & neck, breast, abdomen, and pelvic regions with the nnU‐Net pipeline showing high performance and robustness regardless of anatomical site or imaging modality used.^47^

nnU‐Net involves the use of three different types of parameters: Fixed, rule‐based, and empirical. The fixed parameters remain unchanged when applying nnU‐Net to various datasets and include parameters such as learning rate, optimizer, loss function, architecture template, data augmentation, training, and inference procedures. Once a dataset has been provided, in the correct format, nnU‐Net steps in and determines several rule‐based parameters according to the dataset fingerprint. These include: intensity normalization, image target spacing, as well as image and annotation resampling. nnU‐Net determines the remainder of the rule‐based parameters such as patch size, network topology and batch size for 2D, 3D, and low‐resolution (required for cascade configuration) iteratively based on the available GPU memory. A summary of the nnU‐Net rule‐based parameters for the current study can be found in section A5 of the supplementary material.

**FIGURE 1:**
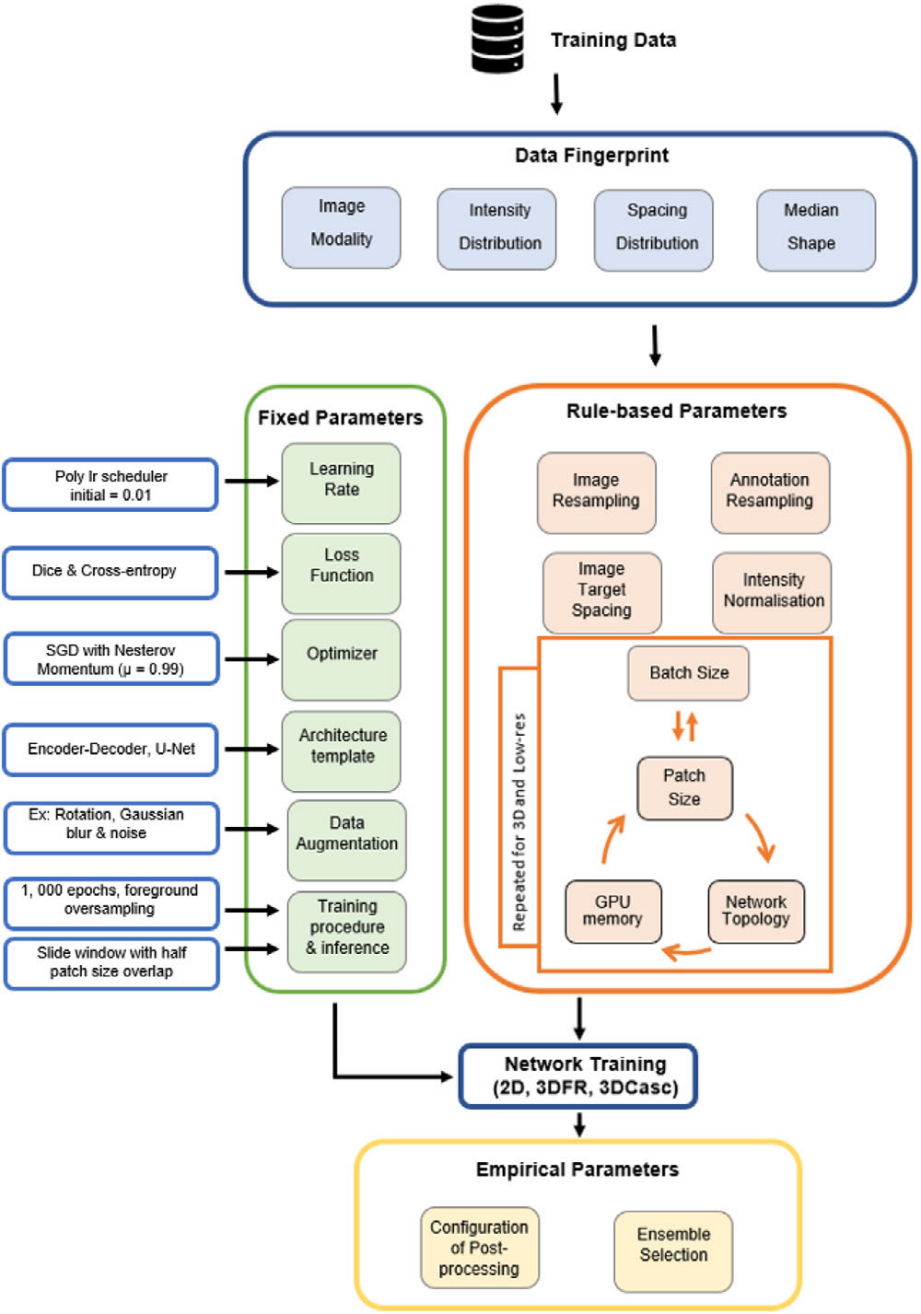
nnU‐Net automated segmentation pipeline.

A summary depicting how the nnU‐Net segmentation pipeline operates as well as certain design and configuration choices, such as learning rate scheduler and loss function is shown in Figure 1. nnU‐Net provides three different trainable U‐Net configurations: a two‐dimensional (2D) U‐Net, 3D full resolution U‐Net (3DFR) and a 3D cascade U‐Net (3DCasc) where the first part is trained on low resolution images followed by refining the segmentation maps at full resolution.

nnU‐Net allows the user to train all configurations using five‐fold cross‐validation, and once completed, nnU‐Net can empirically determine the best performing configuration or ensemble of configurations for the specific task as well as whether or not to apply all‐but‐largest‐component‐suppression post‐processing. A main concern with running ensembles is the significantly high prediction times that can lead to impractical clinical application. For this study, all configurations were trained using five‐fold cross‐validation, where the best single configuration was determined independently using the performance metrics defined in section 2.D, as well as visual inspection of the predicted contours.

The total loss function implemented by nnU‐Net is a combination of the Dice loss function and categorical cross entropy loss function as seen below.

(1) $$\mathrm{Total}\;\mathrm{Loss}=CE_{\mathrm{loss}}+\mathrm{DS}C_{\mathrm{loss}}$$

With the categorical loss function given as:

(2) $$CE_{\mathrm{loss}}=\;\sum_{n\;=\;1}^{N}\sum_{c\;=\;1}^{C}y_{n,c}\log\left(p_{n,c}\right)$$

and the Dice loss function defined as:

(3) $$\mathrm{DS}C_{\mathrm{loss}}=1-\sum_{c\;=\;1}^{C}\frac{\sum_{n\;=\;1}^{N}y_{n,c}p_{n,c}+\varepsilon }{\sum_{n\;=\;1}^{N}p_{n,c}+\sum_{n\;=\;1}^{N}y_{n,c}+\varepsilon }$$

where p_n,c_ is the probability that class label c in pixel n is correct, while y_n,c_ is the one‐hot encoding of the ground truth label for pixel n and the indices n and c iterate over all the pixels and classes, respectively. The ε term avoids the numerical issue of division by zero when either the ground truth mask or prediction segmentation map are empty.

### 2.3 Training

All three configurations were trained using a polynomial learning rate scheduler with initial learning rate of 0.01, the stochastic gradient descent (SGD) optimizer with Nesterov Momentum (μ = 0.99) and trained for 1000 epochs. The 2D U‐Net configuration was trained on the full resolution of the image (512 × 512) with a batch size of 66, while the 3DFR was trained on a batch size of 2 with a patch size of 40 × 256 × 240. The 3DCasc configuration was first trained on downsampled low‐resolution images, with a median image shape 67 × 380 × 380, on a batch size of 2 with a patch size of 40 × 256 × 240 and then further refined at full resolution with the same batch and patch size. Training was carried out on the google colab pro+ server, with access to GPU (NVIDIA Tesla P100) and 52 GB ram.

### 2.4 Performance metrics

The performance of the three generated models (2D, 3DFR, and 3DCasc) were assessed using the 20 test patients. The predicted and manual contours were compared quantitatively using the Dice Score (DSC), Hausdorff Distance (HD), 95 percentile Hausdorff Distance (HD95), Mean Surface Distance (MSD), and Precision Score. Where the DSC focuses on the geometric overlap between the predicted and manual contours, while the HD, HD95, and MSD look at the boundary similarity between the manual and predicted segmentations.

### 2.5 Dosimetric evaluation

All brachytherapy plans were generated using the Oncentra (Elekta, Stockholm, Sweden, version 4.6) treatment planning system according to the OAR dose constraints and HR‐CTV prescribed dose recommended by the American Brachytherapy Society (ABS)^50^ and GEC‐ESTRO^50^, ^51^, ^52^ guidelines. Assuming α/β = 10, a prescribed dose (EB + brachy) of D_90_ > 80 Gy_EQD2_ for the HR‐CTV and assuming an α/β = 3, the maximum dose (D_2cc_) to the bladder and rectum (EB + brachy) of 90 Gy_EQD2_ and 75 Gy_EQD2_ respectively. To evaluate the dosimetric differences between the manual contours and predicted contours, the predicted segmentations were converted to DICOM before importing into the planning system and the original dose distribution was overlaid and cumulative dose volume histograms (DVHs) generated. The Dosimetric discrepancies of D_90%_ for HR‐CTV and D_2cc_ for the OARS were evaluated and compared with the DVH statistics generated from the manual contours.

### 2.6 Clinician assessment

Performance and dosimetric evaluation provided a good indication of a model's accuracy; however, these metrics alone were not sufficient and required input from clinicians to properly assess the clinical acceptability of the generated patient contours. The OARs and HR‐CTVs from each of the 20 test patients were evaluated independently by three different ROs, scoring each patient's bladder, rectum, and HR‐CTV on a scale of 1 to 4 where; 1 indicates reject completely, 2 major revisions, 3 minor revisions before clinically acceptable, and 4 indicating clinically acceptable as is. Contours scored with a 4, also included ones where ROs would have preferred some small adjustments but were not necessary for the contours to be deemed clinically acceptable. For any contours scoring a 3 or lower, the ROs indicated the time required to adjust the contours to the level of clinical acceptability. These times, along with the model prediction times were compared to the average time taken for ROs to manually contour all the OARs and HR‐CTV.

## 3 RESULTS

### 3.1 Model comparisons

The mean ± standard deviation of the performance metrics (DSC, HD, HD95, MSD, and precision) between the manual contours and predicted contours for all three generated nnU‐Net configurations (2D, 3DFR, and 3DCasc) are given in Table 1. The best performing configurations were 3DFR and 3DCasc with the mean DSC over all OARs and HR‐CTVs for both configurations being around 0.85. The mean HD, HD95, and MSD for the 3DFR and 3DCasc configurations over all OARs and HR‐CTVs were approximately 9.9/10.1 mm, 4.8/4.9 mm, and 1.48/1.54 mm, respectively.

**TABLE 1:** Mean DSCs, MSDs (mm), HDs (in mm), and HD95s (in mm) between the manual and predicted contours for all three configurations

Contour	Model	DSC	HD (mm)	HD95 (mm)	MSD (mm)	Precision
Bladder	2D	0.87 ± 0.05	18.44 ± 0.91	5.71 ± 3.26	1.53 ± 0.77	0.86 ± 0.08
	3DFR	0.92 ± 0.04	7.52 ± 3.13	3.00 ± 1.09	0.84 ± 0.30	0.91 ± 0.05
	3DCasc	0.91 ± 0.04	8.39 ± 3.49	3.26 ± 1.06	0.94 ± 0.33	0.91 ± 0.05
Rectum	2D	0.80 ± 0.05	15.48 ± 5.55	6.17 ± 2.25	1.68 ± 0.52	0.78 ± 0.05
	3DFR	0.84 ± 0.04	13.78 ± 4.62	5.25 ± 1.78	1.36 ± 0.43	0.84 ± 0.04
	3DCasc	0.84 ± 0.04	13.48 ± 3.86	5.36 ± 1.65	1.39 ± 0.41	0.83 ± 0.04
HR CTV	2D	0.78 ± 0.06	11.69 ± 3.22	6.66 ± 2.03	2.64 ± 0.92	0.77 ± 0.11
	3DFR	0.81 ± 0.05	8.48 ± 1.78	6.03 ± 2.01	2.23 ± 0.75	0.80 ± 0.09
	3DCasc	0.81 ± 0.05	8.71 ± 2.04	6.14 ± 2.01	2.30 ± 0.72	0.80 ± 0.09

The predicted contours from all three configurations were also visually inspected and the 3DFR configuration was found to provide slightly better predictions when it came to the larger bladder volumes.

### 3.2 Performance results

**FIGURE 2:**
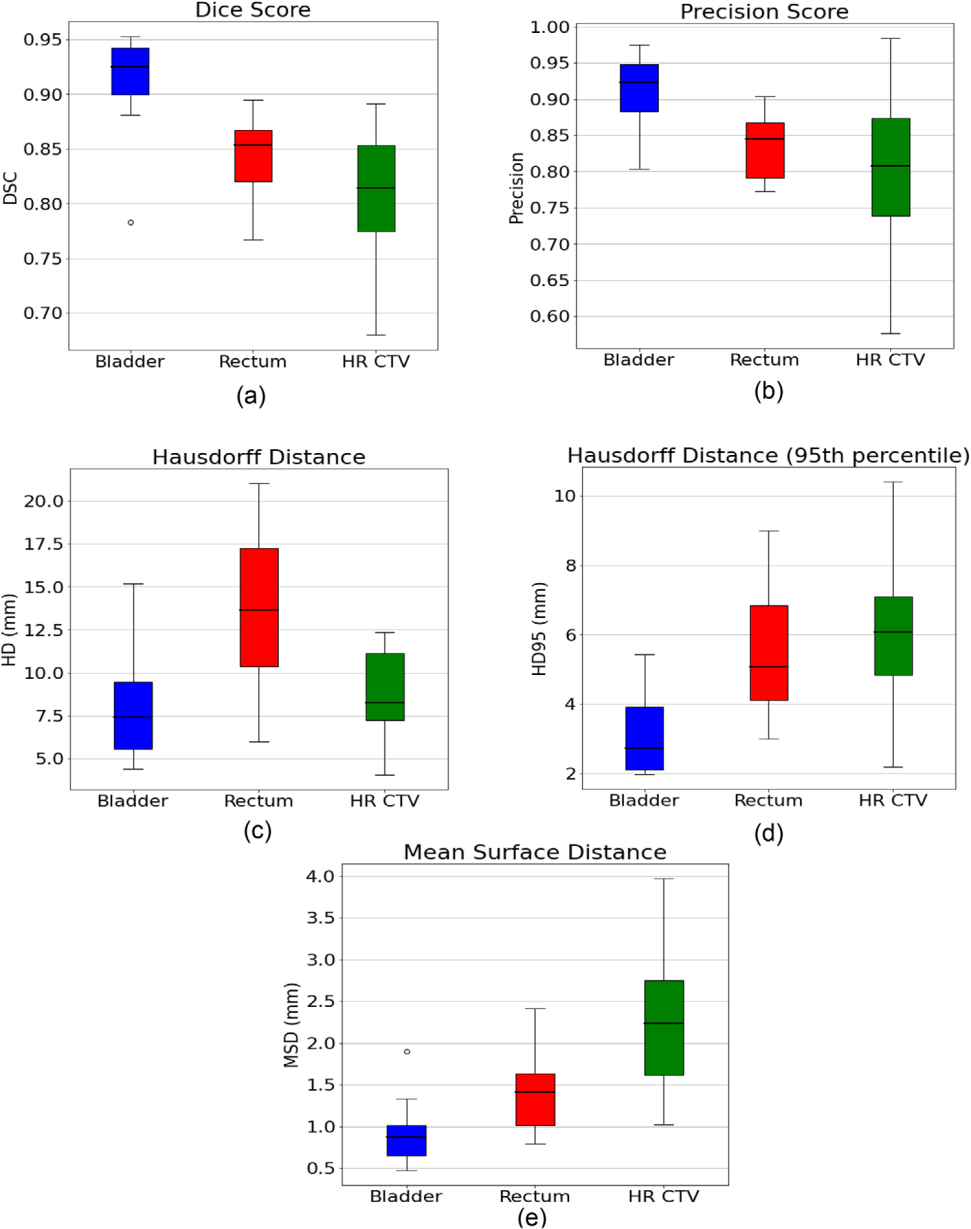
Box plots showing the performance metrics of the bladder, rectum, and HR CTV for the 3DFR nnU‐Net configuration. (a) DSC, (b) precision score, (c) HD, (d) HD95, and (e) the MSD.

**FIGURE 3:**
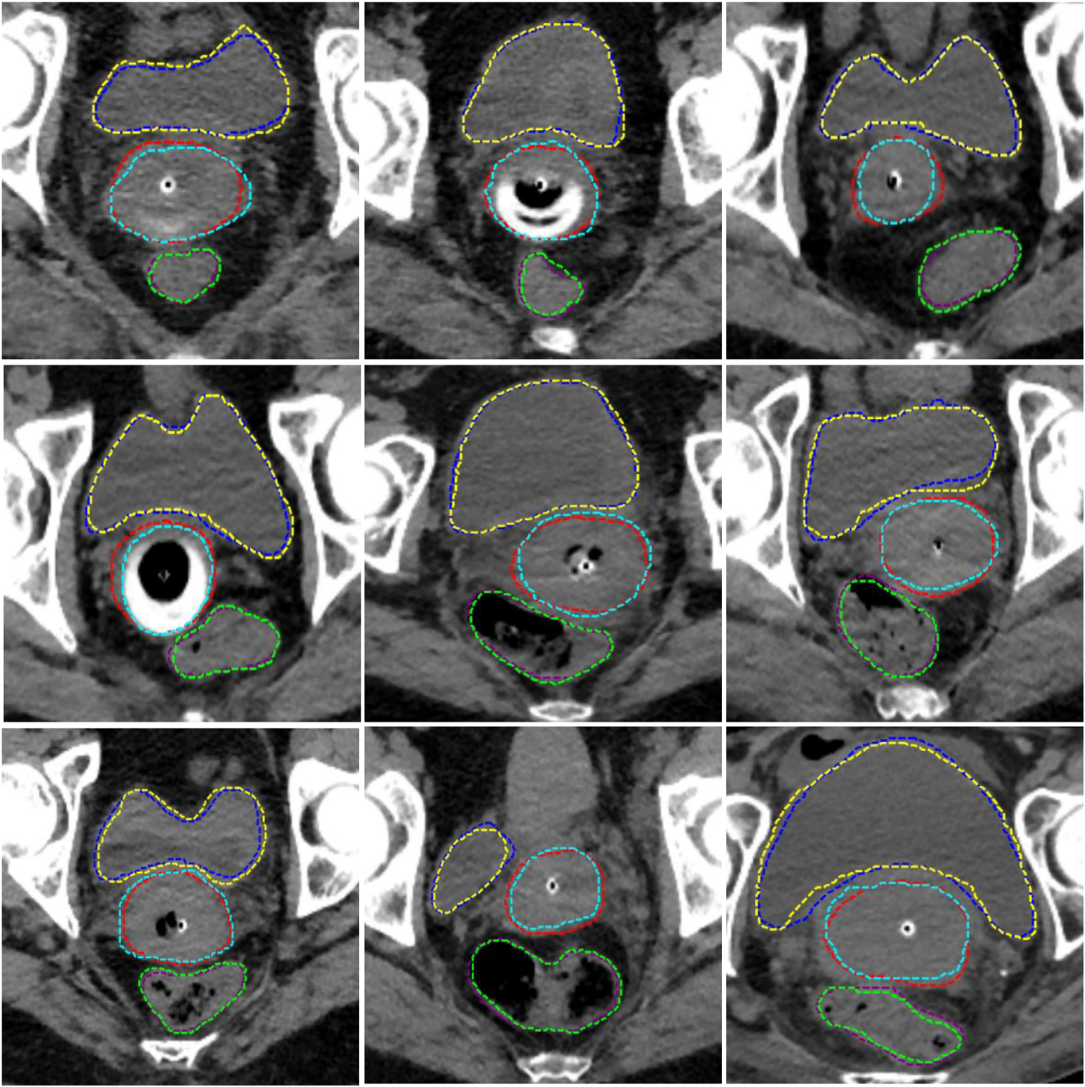
Examples showing comparisons between manual and predicted contours. Blue = predicted bladder, Yellow = ground truth bladder, Purple = predicted rectum, Green = ground truth rectum, Red = predicted HR CTV, and Light Blue = ground truth HR CTV.

The overall boxplots of the DSC, HD, HD95, MSD, and precision scores for each structure of the 3DFR configuration is shown in Figure 2. Almost all predicted contours had performance metrics that were distributed within a certain range, with only a single low DSC and high MSD outlier in the boxplots for the bladder contours. Examples comparing the manual contours to those predicted by the 3DFR nnU‐Net configuration are shown in Figure 3.

### 3.3 Dosimetric evaluation

**FIGURE 4:**
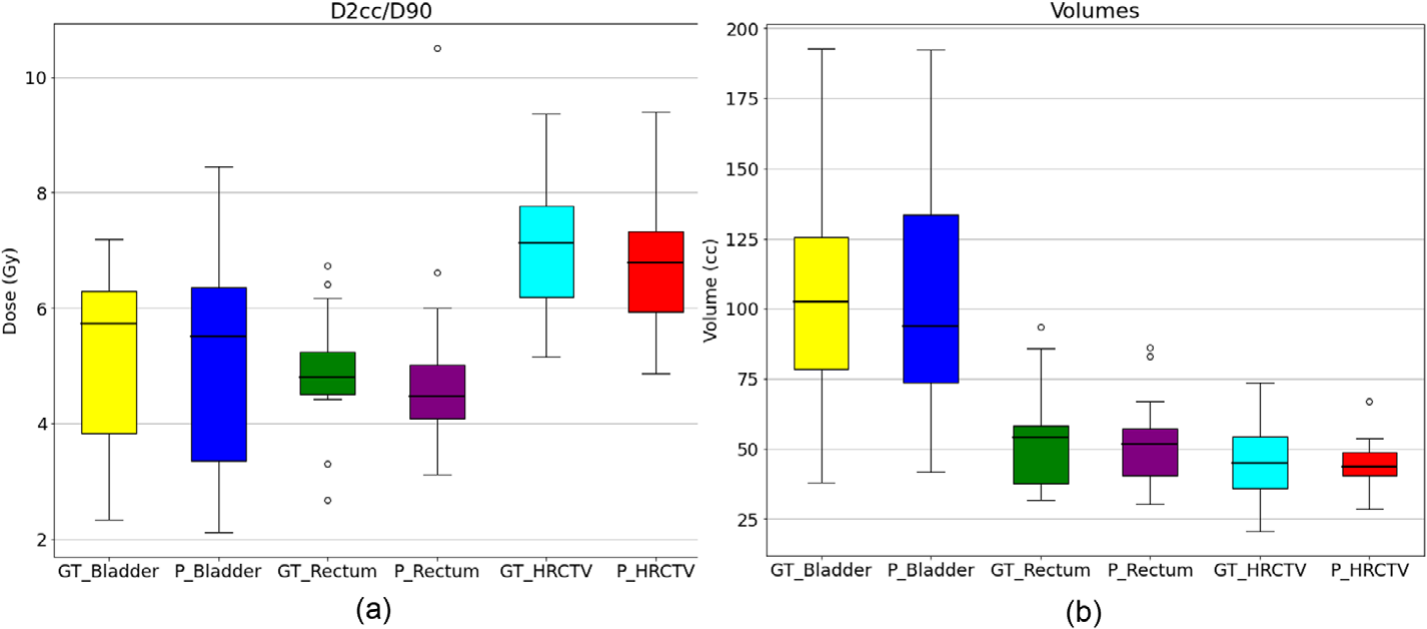
(a) DVH parameters and (b) volumes for the ground truth and predicted contours. GT = Ground Truth and P = Predicted.

**FIGURE 5:**
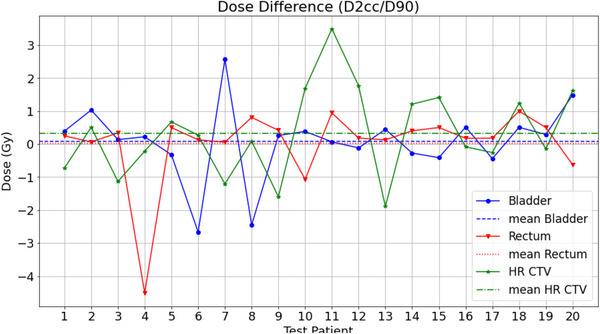
Plot of the dose difference in the DVH parameters. D2cc for OARs and D90 for the HR CTV.

The dosimetric accuracy of the 3DFR nnU‐Net predictions was investigated by looking at the DVH parameter D_2cc_ for the OARs and D_90%_ for the HR CTV, as well the total volume (in cm^3^ or cc) of the OARs and HR CTVs. Boxplots showing the DVH parameters and volumes of all 20 test patients for the manual and predicted contours are given in Figure 4. The dose differences for the DVH parameters (D_2cc_/D_90_) for each test patient is shown in Figure 5. The mean dose and volume differences over all OARs and HR CTVs was 0.1 ± 1.2 Gy and 1.1 ± 7.1 cm^3^, respectively.

A summary of the mean ± standard deviation of the DVH parameters and volumes for the manual and predicted contours are shown in Table 2, along with the mean ± standard deviation of the DVH dose parameters and volume differences.

**TABLE 2:** Results of the dosimetric and volume comparison of the bladder, rectum, and HR CTV for the manual and predicted contours

Contour	Manual		Predicted		Mean Change	
	D2cc/D90 (Gy)	vol (cm^3^)	D2cc/D90 (Gy)	vol (cm^3^)	∆D2cc/90% (Gy)	∆vol (cm^3^)
Bladder	5.2 ± 1.5	108.6 ± 45.7	5.1 ± 1.8	107.4 ± 47.5	0.08 ± 1.14	1.3 ± 7.3
Rectum	4.9 ± 1.0	53.0 ± 16.8	4.9 ± 1.6	52.3 ± 14.5	0.02 ± 1.17	0.7 ± 6.2
HR CTV	7.1 ± 1.2	45.6 ± 12.8	6.8 ± 1.2	44.1 ± 8.9	0.33 ± 1.33	1.5 ± 8.0

All values reported as mean ± standard deviation.

### 3.4 Clinician reviews

**FIGURE 6:**
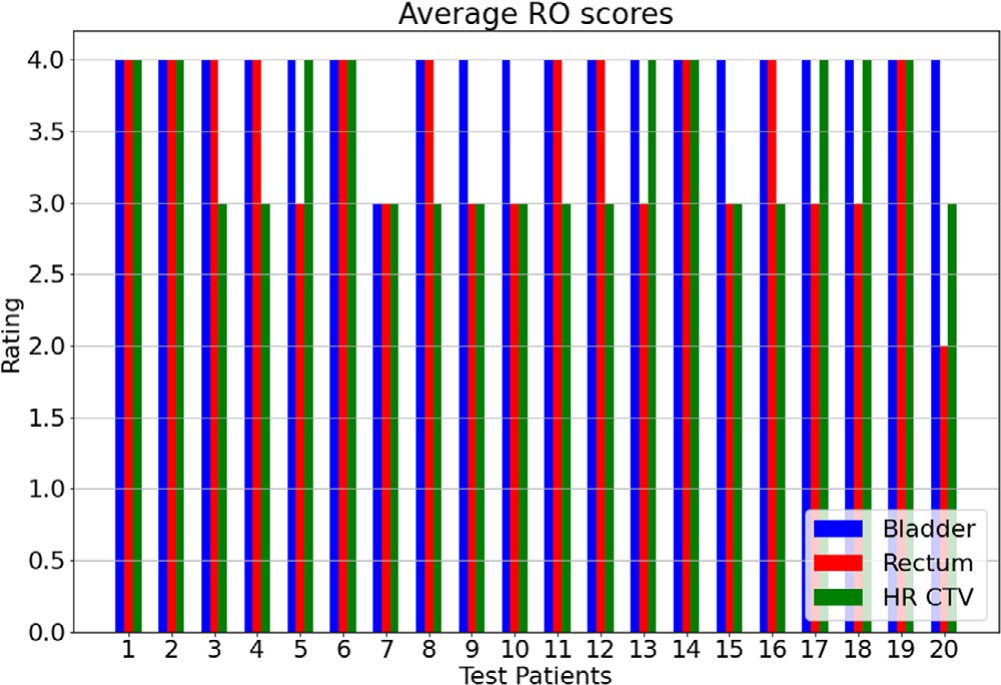
Average RO reviews of the predicted bladder, rectum, and HR CTV contours for all 20 test patients.

The average scores from all three of the ROs reviews of the predicted bladder, rectum, and HR CTVs for all 20 test patients are shown in Figure 6. Overall, OARs and HR CTV combined, 65% of the predicted contours were suitable for clinical use, while 33% required minor adjustments before being clinically accepted, the remaining 2% required major revisions and 0% were rejected completely. A summary of the average review scores for the bladder rectum and HR CTV is given in Table 3.

**TABLE 3:** Summary of the average RO review scores for the predicted contours of the bladder, rectum, and HR CTV for the 20 test patients

Contour	Accept	Minor edits	Major edits	Reject
Bladder	95 % (19)	5 % (1)	0 % (0)	0 % (0)
Rectum	55 % (11)	40 % (8)	5 % (1)	0 % (0)
HR CTV	45 % (9)	55 % (11)	0 % (0)	0 % (0)

For patient contours that scored a 3 or lower, the ROs recorded the time needed to make the necessary corrections to the contours. The mean editing time, along with the mean manual contouring time and model prediction time are given in Table 4. The mean time taken for the auto‐contouring system to delineate the OARs and HR CTV is substantially less than the mean manual contouring time.

**TABLE 4:** Results of manual contouring time, model prediction time and editing times

	Manual	Prediction	Edits
Times (min)	14.0 ± 3.3	1.607 ± 0.004	2.1 ± 1.0

All values reported as mean ± standard deviation.

## 4 DISCUSSION

With only a few lines of code, we successfully implemented the self‐configuring nnU‐Net for the automatic segmentation of the OARs and HR CTV in HDR cervical brachytherapy. Three nnU‐Net configurations were trained (2D, 3DFR, and 3DCasc) with a total of 64 training CTs and 16 validation CTs. All three configurations were tested on the remaining 20 CT scans, where the 3DFR and 3DCasc models provided substantially better performance with the 3DFR providing better generalization over the larger bladder volumes when inspected visually. We demonstrated that the nnU‐Net can produce clinically acceptable contours for both the OARs and HR CTV, with significant time saving possible when applying the automatic contouring system.

### 4.1 Quantitative results

All three nnU‐Net configurations were assessed using the performance metrics described in Section 2.C with 3DFR and 3DCasc models significantly outperforming the 2D model (*P* < 0.002) over all OAR and HR CTV contours combined (Table 1). There were no statistical differences between the 3DFR and 3DCasc models (*P* > 0.863); however, after visual inspection, the 3DFR model provided slightly better generalization when it came to larger bladder volumes. The 3DFR model predicted bladder, rectum, and HR CTV contours with mean DSC/HD95/MSDs of 0.92/3.0 mm/0.8 mm, 0.84/5.4 mm/1.4 mm, and 0.81/6.0 mm/2.2 mm, respectively. Among all the contours, it was found that the bladder provided the highest DSC and precision scores, as well as the lowest distance metrics (HD, HD95, and MSD). The largest variation in performance metric results was found to be the HR CTV. This can be attributed to the fact that HR CTVs do not have a clearly visible anatomical boundary or edge, especially in CT imaging, which can cause inter‐ and intra‐observer variability in the ground truth contours as well as a drop in model performance for the HR‐CTV when compared to the OARs with more clearly defined edges. A comparison of the 3DFR model performance metrics to those found in previous work is shown in Table 5.

**TABLE 5:** Comparison of performance metric between the current study and previous work

Studies	HR CTV	Bladder	Rectum
Zhang et al.^40^	DSC: 83 ± 4%	DSC: 87 ± 3%	DSC: 82 ± 5%
Method: DSD‐UNet	HD: 8.1 ± 2.3 mm	HD: 12.1 ± 4.0 mm	HD: 9.2 ± 4.6 mm
Wong et al.^41^	DSC: 71%	DSC: 92%	DSC: 76%
	HD95: 9.32 mm	HD95: 3.5 mm	HD95: 18.4 mm (rectum + sigmoid)
Mohammadi et al.^42^		DSC: 96 ± 4%	DSC: 97 ± 2%
Method: 2D ResU‐Net		HD95: 2.3 ± 3.4 mm	HD95: 1.4 ± 1.4 mm
Yoganathan et al.^43^	DSC: 85 ± 6%	DSC: 90 ± 5%	DSC: 76 ± 7%
2.5D‐InRN	HD95: 4.9 ± 2.2 mm	HD95: 6.3 ± 3.4 mm	HD95: 8.2 ± 4.1 mm
Cao et al.^44^	DSC: 76 ± 6%		
Method: Asym dual path CNN	HD95: 6.0 ± 1.7 mm		
Current Study	DSC: 81 ± 5 %	DSC: 92 ± 4 %	DSC: 84 ± 4 %
3DFR nnU‐Net	HD: 8.5 ± 2.5 mm	HD: 7.5 ± 3.1 mm	HD: 13.8 ± 4.6 mm
	HD95: 6.0 ± 2.0 mm	HD95: 3.0 ± 1.1 mm	HD95: 5.3 ± 1.8 mm

The performance of the 3DFR nnU‐Net configuration compared well to work done in previous studies, where complex model architectures were designed specific to the task of HDR cervical brachytherapy contouring. 3DFR managed to outperform two of the studies when it came to bladder and rectum contouring, beaten only by the 2D ResU‐Net by Mohammadi et al.^42^ However, this study did not look at the segmentation of the HR CTV, included a larger dataset and contained CT scans where strict bladder and rectal filling protocols were followed, a limitation of this study. The current work provided HR CTV contour performance comparable to those found in other studies.

### 4.2 Dosimetric accuracy

Dosimetric evaluation between the predicted and manual contours was carried out for all 20 test patients. There were no statistically significant differences in the mean D_2cc_/D_90%_ or volumes for the bladder, rectum, and HR CTV (all *P* > 0.8). The largest mean change in DVH parameter was for the HR CTV, with a mean change in D_90%_ of 0.33 Gy and mean change in volume of 1.5 cm^3^. The mean change in D_2cc_ and volume for the bladder and rectum (Table 2) showed better accuracy than those found in other studies, where Mohammadi et al.^42^ had mean changes in D_2cc_/volume of −0.5 Gy/34 cm^3^ and 0.3 Gy/17 cm^3^ for the bladder and rectum respectively. The largest difference in D_2cc_ of −4.5 Gy (Figure 5) was observed for a rectum contour of a single test patient, this test patient coincides with the lowest DSC value (0.77) and one of the highest HD values (20 mm) obtained for the predicted rectum contours.

### 4.3 Clinical acceptability

There was an average acceptance rate 65% over all the OARs and HR CTVs for the 20 test patients, with an average acceptance rate of 95% (19 patients) and 55% (11 patients) for the bladder and rectum, respectively. Approximately 40% (eight patients) for the rectum and 55% (11 patients) for the HR CTV required very minor revisions before being clinically acceptable. Most of the minor revisions for the rectum were the superior border of the rectum, where the model struggled slightly with distinguishing the rectum from sections of the bowel or sigmoid (see section A4 of supplementary material). The minor revisions for the HR CTV were a combination of either incomplete contours at the superior borders or at the inferior border, where too much of the ring was included in the contour. Based on the average scores for the bladder, minor revisions were only required for one patient, where the model failed to accurately determine the bladder wall edge.

The average time taken for ROs to manually contour out the OARs and HR CTV was around 14 min, while the average model prediction time was 1.6 min and average editing time of approximately 2.1 min (for those that required minor revisions before being clinically acceptable). The results show that application of the auto‐contouring system, on average, has the capability to substantially reduce the overall required contouring time thereby allowing for a more efficient planning workflow by alleviating the bottleneck experienced at the contouring stage.

This study faced several limitations such as small dataset size, no consistent bladder or rectum preparation and no contrast agent. Over and above these limitations, this study was also hindered by inter observer variations in the manual contours, used for training, as well as inter observer variations between the three ROs scoring the generated contours. In addition, it is acknowledged that CT‐based brachytherapy contours are inferior in accuracy to MRI‐based contours.

## 5 CONCLUSION

We have demonstrated that with only a few lines of code and relatively small patient dataset, one can develop an accurate and robust auto‐segmentation model that provides clinically acceptable contours of the OARs and HR CTV in T‐R HDR cervical brachytherapy, with no significant dose difference between manual and predicted contours. We have shown that an auto‐contouring system can significantly reduce the required contouring time thereby providing a more efficient planning workflow. This is ideally suited to a resource‐constrained oncology department using CT‐based image guided brachytherapy.

## CONFLICT OF INTEREST STATEMENT

Ethics approval was obtained from the University of Stellenbosch Health Research Ethics Committee (HREC) with project ID 24418 and ethics reference number S22/01/013. The authors have no conflict of interest to disclose.

The nnU‐Net code developed by Isensee et al^46^ can be found at: https://github.com/MIC‐DKFZ/nnUNet.

## Supporting information

Supporting Information
