# Supplementary material for: Self‐configuring nnU‐Net for automatic delineation of the organs at risk and target in high‐dose rate cervical brachytherapy, a low/middle‐income country's experience
Source: J Appl Clin Med Phys. 2023 Apr 12;24(8):e13988. doi: 10.1002/acm2.13988 (PMC10402684; doi:10.1002/acm2.13988)
Supplement: Supplementary file 1 — Supporting Information [file ACM2-24-e13988-s001.docx]

**SUPPLEMENTARY MATERIAL**

**A1: Performance of 2D nnU-Net configuration**

Table A1-1. Performance Metrics for the bladder of all 20 test patients for the 2D U-Net configuration.

| Test Pt | DSC | HD (mm) | HD95 (mm) | MSD (mm) | Precision |
| --- | --- | --- | --- | --- | --- |
| 1 | 0.91 | 6.27 | 2.88 | 0.97 | 0.96 |
| 2 | 0.79 | 23.11 | 16.24 | 3.71 | 0.88 |
| 3 | 0.93 | 20.12 | 2.49 | 0.76 | 0.93 |
| 4 | 0.92 | 6.00 | 2.49 | 0.80 | 0.97 |
| 5 | 0.87 | 15.43 | 5.69 | 1.64 | 0.92 |
| 6 | 0.92 | 5.59 | 2.61 | 0.80 | 0.96 |
| 7 | 0.87 | 13.42 | 4.67 | 1.41 | 0.95 |
| 8 | 0.74 | 22.46 | 9.76 | 2.87 | 0.90 |
| 9 | 0.91 | 25.13 | 6.62 | 1.48 | 0.95 |
| 10 | 0.86 | 30.94 | 5.61 | 1.58 | 0.92 |
| 11 | 0.84 | 40.48 | 6.92 | 1.90 | 0.93 |
| 12 | 0.91 | 10.23 | 4.88 | 1.26 | 0.94 |
| 13 | 0.85 | 10.00 | 5.06 | 1.40 | 0.93 |
| 14 | 0.94 | 6.85 | 3.24 | 0.75 | 0.95 |
| 15 | 0.91 | 35.65 | 6.94 | 1.65 | 0.93 |
| 16 | 0.90 | 9.22 | 4.53 | 1.08 | 0.95 |
| 17 | 0.91 | 25.29 | 2.49 | 0.83 | 0.95 |
| 18 | 0.87 | 35.27 | 5.43 | 1.48 | 0.93 |
| 19 | 0.80 | 14.34 | 9.10 | 2.67 | 0.91 |
| 20 | 0.82 | 13.02 | 6.61 | 1.58 | 0.92 |

Table A1-2. Performance Metrics for the rectum of all 20 test patients for the 2D U-Net configuration.

| Test Pt | DSC | HD (mm) | HD95 (mm) | MSD (mm) | Precision |
| --- | --- | --- | --- | --- | --- |
| 1 | 0.80 | 10.05 | 5.44 | 1.58 | 0.87 |
| 2 | 0.84 | 18.79 | 3.90 | 1.25 | 0.89 |
| 3 | 0.80 | 16.71 | 5.19 | 1.34 | 0.81 |
| 4 | 0.72 | 27.00 | 8.66 | 2.26 | 0.84 |
| 5 | 0.87 | 9.25 | 4.59 | 1.41 | 0.91 |
| 6 | 0.82 | 11.71 | 6.00 | 1.64 | 0.86 |
| 7 | 0.84 | 14.83 | 4.09 | 1.26 | 0.90 |
| 8 | 0.81 | 11.51 | 4.82 | 1.62 | 0.88 |
| 9 | 0.85 | 13.12 | 4.64 | 1.40 | 0.88 |
| 10 | 0.81 | 10.47 | 5.36 | 1.48 | 0.83 |
| 11 | 0.70 | 22.56 | 12.62 | 2.99 | 0.79 |
| 12 | 0.79 | 9.15 | 4.84 | 1.38 | 0.81 |
| 13 | 0.86 | 9.95 | 4.22 | 1.12 | 0.89 |
| 14 | 0.82 | 18.75 | 5.62 | 1.50 | 0.85 |
| 15 | 0.76 | 19.15 | 9.19 | 2.45 | 0.71 |
| 16 | 0.84 | 11.18 | 5.00 | 1.25 | 0.87 |
| 17 | 0.84 | 17.94 | 6.98 | 1.50 | 0.76 |
| 18 | 0.72 | 26.84 | 8.29 | 2.48 | 0.80 |
| 19 | 0.84 | 12.84 | 5.06 | 1.35 | 0.86 |
| 20 | 0.74 | 17.83 | 8.96 | 2.33 | 0.73 |

Table A1-3. Performance Metrics for the HR CTV of all 20 test patients for the 2D U-Net configuration.

| Test Pt | DSC | HD (mm) | HD95 (mm) | MSD (mm) | Precision |
| --- | --- | --- | --- | --- | --- |
| 1 | 0.78 | 12.88 | 7.60 | 3.16 | 0.93 |
| 2 | 0.72 | 12.95 | 9.64 | 3.54 | 0.90 |
| 3 | 0.83 | 6.83 | 4.44 | 1.59 | 0.93 |
| 4 | 0.82 | 12.39 | 5.00 | 2.00 | 0.94 |
| 5 | 0.81 | 9.37 | 6.08 | 2.47 | 0.94 |
| 6 | 0.83 | 6.96 | 3.46 | 1.46 | 0.95 |
| 7 | 0.83 | 10.74 | 4.12 | 1.79 | 0.93 |
| 8 | 0.66 | 12.62 | 10.22 | 4.02 | 0.91 |
| 9 | 0.79 | 11.04 | 5.63 | 2.48 | 0.94 |
| 10 | 0.71 | 12.39 | 8.90 | 3.70 | 0.90 |
| 11 | 0.67 | 18.75 | 9.26 | 4.38 | 0.90 |
| 12 | 0.69 | 15.30 | 9.48 | 4.11 | 0.86 |
| 13 | 0.83 | 11.46 | 5.53 | 1.90 | 0.93 |
| 14 | 0.74 | 13.30 | 8.03 | 3.00 | 0.89 |
| 15 | 0.77 | 16.88 | 7.01 | 2.67 | 0.89 |
| 16 | 0.78 | 10.92 | 6.59 | 2.49 | 0.94 |
| 17 | 0.85 | 6.08 | 4.59 | 1.64 | 0.94 |
| 18 | 0.76 | 11.46 | 6.24 | 2.14 | 0.94 |
| 19 | 0.81 | 13.28 | 6.42 | 2.57 | 0.94 |
| 20 | 0.84 | 8.24 | 5.01 | 1.60 | 0.92 |

**A2: Performance of 3DCasc nnU-Net configuration**

Table A2-1. Performance Metrics for the bladder of all 20 test patients for the 3DCasc U-Net configuration.

| Test Pt | DSC | HD (mm) | HD95 (mm) | MSD (mm) | Precision |
| --- | --- | --- | --- | --- | --- |
| 1 | 0.93 | 4.88 | 2.11 | 0.70 | 0.98 |
| 2 | 0.94 | 11.30 | 3.23 | 0.97 | 0.98 |
| 3 | 0.94 | 6.03 | 2.70 | 0.75 | 0.97 |
| 4 | 0.94 | 4.96 | 2.09 | 0.68 | 0.97 |
| 5 | 0.91 | 15.31 | 4.07 | 1.06 | 0.96 |
| 6 | 0.95 | 4.39 | 1.98 | 0.47 | 0.97 |
| 7 | 0.89 | 12.62 | 4.15 | 1.24 | 0.96 |
| 8 | 0.78 | 12.51 | 5.64 | 1.96 | 0.94 |
| 9 | 0.93 | 11.35 | 4.23 | 1.00 | 0.96 |
| 10 | 0.90 | 7.87 | 3.11 | 1.01 | 0.94 |
| 11 | 0.89 | 6.61 | 3.17 | 0.97 | 0.97 |
| 12 | 0.95 | 6.61 | 2.86 | 0.78 | 0.97 |
| 13 | 0.88 | 7.23 | 3.70 | 0.97 | 0.96 |
| 14 | 0.95 | 6.31 | 2.49 | 0.56 | 0.97 |
| 15 | 0.92 | 15.05 | 4.96 | 1.34 | 0.92 |
| 16 | 0.89 | 10.82 | 4.84 | 1.23 | 0.96 |
| 17 | 0.92 | 6.16 | 2.40 | 0.75 | 0.95 |
| 18 | 0.91 | 5.33 | 2.78 | 0.89 | 0.95 |
| 19 | 0.94 | 6.00 | 2.38 | 0.77 | 0.97 |
| 20 | 0.92 | 6.55 | 2.38 | 0.66 | 0.95 |

Table A2-2. Performance Metrics for the rectum of all 20 test patients for the 3DCasc configuration.

| Test Pt | DSC | HD (mm) | HD95 (mm) | MSD (mm) | Precision |
| --- | --- | --- | --- | --- | --- |
| 1 | 0.85 | 12.27 | 5.95 | 1.40 | 0.90 |
| 2 | 0.86 | 8.34 | 4.25 | 1.21 | 0.89 |
| 3 | 0.85 | 10.41 | 3.68 | 0.98 | 0.85 |
| 4 | 0.76 | 17.51 | 6.77 | 1.70 | 0.88 |
| 5 | 0.90 | 12.87 | 3.67 | 1.14 | 0.93 |
| 6 | 0.87 | 12.54 | 5.53 | 1.26 | 0.88 |
| 7 | 0.85 | 14.83 | 4.40 | 1.23 | 0.89 |
| 8 | 0.80 | 11.30 | 4.97 | 1.63 | 0.85 |
| 9 | 0.86 | 18.36 | 6.03 | 1.51 | 0.90 |
| 10 | 0.81 | 11.18 | 6.28 | 1.68 | 0.84 |
| 11 | 0.84 | 15.61 | 7.09 | 1.38 | 0.89 |
| 12 | 0.86 | 7.67 | 3.78 | 0.95 | 0.86 |
| 13 | 0.89 | 8.90 | 3.02 | 0.84 | 0.92 |
| 14 | 0.83 | 15.70 | 5.68 | 1.48 | 0.85 |
| 15 | 0.76 | 20.12 | 9.96 | 2.60 | 0.74 |
| 16 | 0.87 | 8.00 | 3.09 | 0.83 | 0.90 |
| 17 | 0.89 | 17.38 | 5.12 | 1.15 | 0.87 |
| 18 | 0.79 | 19.62 | 6.76 | 1.91 | 0.86 |
| 19 | 0.83 | 12.84 | 6.19 | 1.55 | 0.87 |
| 20 | 0.84 | 14.12 | 5.06 | 1.43 | 0.83 |

Table A2-3. Performance Metrics for the HR CTV of all 20 test patients for the 3DCasc configuration.

| Test Pt | DSC | HD (mm) | HD95 (mm) | MSD (mm) | Precision |
| --- | --- | --- | --- | --- | --- |
| 1 | 0.80 | 8.00 | 8.00 | 3.11 | 0.93 |
| 2 | 0.79 | 10.78 | 9.03 | 2.72 | 0.93 |
| 3 | 0.79 | 8.52 | 6.07 | 2.08 | 0.91 |
| 4 | 0.88 | 5.67 | 3.48 | 1.23 | 0.97 |
| 5 | 0.81 | 8.00 | 5.80 | 2.32 | 0.94 |
| 6 | 0.81 | 6.03 | 4.06 | 1.74 | 0.95 |
| 7 | 0.84 | 6.00 | 4.00 | 1.86 | 0.96 |
| 8 | 0.67 | 13.21 | 11.27 | 4.03 | 0.93 |
| 9 | 0.84 | 9.85 | 5.66 | 1.98 | 0.95 |
| 10 | 0.75 | 8.74 | 8.00 | 3.38 | 0.91 |
| 11 | 0.77 | 8.44 | 6.00 | 2.95 | 0.93 |
| 12 | 0.77 | 11.22 | 8.90 | 3.16 | 0.88 |
| 13 | 0.83 | 8.15 | 4.37 | 2.14 | 0.96 |
| 14 | 0.85 | 9.37 | 6.19 | 1.73 | 0.92 |
| 15 | 0.76 | 9.85 | 6.06 | 2.40 | 0.92 |
| 16 | 0.87 | 9.85 | 4.44 | 1.34 | 0.95 |
| 17 | 0.84 | 6.08 | 5.19 | 1.86 | 0.93 |
| 18 | 0.75 | 11.56 | 5.58 | 2.27 | 0.94 |
| 19 | 0.86 | 6.85 | 4.23 | 1.83 | 0.95 |
| 20 | 0.82 | 8.12 | 6.44 | 1.94 | 0.91 |

**A3: Performance of 3DFR nnU-Net configuration**

Table A3-1. Performance Metrics for the bladder of all 20 test patients for the 3DFR U-Net configuration.

| Test Pt | DSC | HD (mm) | HD95 (mm) | MSD (mm) | Precision |
| --- | --- | --- | --- | --- | --- |
| 1 | 0.93 | 4.49 | 2.11 | 0.69 | 0.98 |
| 2 | 0.94 | 11.63 | 3.36 | 1.06 | 0.97 |
| 3 | 0.95 | 5.67 | 2.11 | 0.56 | 0.98 |
| 4 | 0.94 | 4.86 | 2.00 | 0.65 | 0.97 |
| 5 | 0.93 | 7.67 | 2.51 | 0.82 | 0.97 |
| 6 | 0.95 | 4.39 | 1.98 | 0.47 | 0.97 |
| 7 | 0.89 | 13.39 | 4.59 | 1.33 | 0.96 |
| 8 | 0.78 | 9.09 | 5.43 | 1.90 | 0.94 |
| 9 | 0.93 | 10.57 | 3.89 | 0.93 | 0.96 |
| 10 | 0.90 | 8.52 | 3.68 | 1.01 | 0.94 |
| 11 | 0.89 | 7.92 | 3.60 | 0.98 | 0.96 |
| 12 | 0.95 | 6.31 | 2.78 | 0.74 | 0.96 |
| 13 | 0.88 | 7.09 | 4.01 | 1.00 | 0.97 |
| 14 | 0.95 | 6.31 | 2.47 | 0.57 | 0.96 |
| 15 | 0.92 | 15.21 | 4.84 | 1.25 | 0.93 |
| 16 | 0.89 | 10.57 | 4.80 | 1.20 | 0.96 |
| 17 | 0.93 | 8.00 | 2.11 | 0.65 | 0.97 |
| 18 | 0.91 | 5.26 | 2.68 | 0.93 | 0.94 |
| 19 | 0.95 | 4.39 | 2.09 | 0.55 | 0.98 |
| 20 | 0.92 | 7.11 | 2.38 | 0.71 | 0.95 |

Table A3-2. Performance Metrics for the rectum of all 20 test patients for the 3DFR configuration.

| Test Pt | DSC | HD (mm) | HD95 (mm) | MSD (mm) | Precision |
| --- | --- | --- | --- | --- | --- |
| 1 | 0.85 | 9.76 | 4.84 | 1.29 | 0.91 |
| 2 | 0.86 | 7.88 | 4.12 | 1.25 | 0.89 |
| 3 | 0.87 | 8.83 | 3.00 | 0.85 | 0.87 |
| 4 | 0.77 | 20.14 | 6.86 | 1.65 | 0.88 |
| 5 | 0.87 | 17.22 | 7.22 | 1.62 | 0.91 |
| 6 | 0.90 | 13.97 | 4.63 | 0.97 | 0.90 |
| 7 | 0.86 | 11.38 | 3.48 | 1.02 | 0.89 |
| 8 | 0.81 | 11.60 | 5.96 | 1.66 | 0.86 |
| 9 | 0.86 | 17.43 | 6.01 | 1.49 | 0.88 |
| 10 | 0.80 | 10.59 | 6.24 | 1.74 | 0.84 |
| 11 | 0.83 | 18.89 | 9.00 | 1.60 | 0.87 |
| 12 | 0.86 | 9.15 | 4.11 | 0.94 | 0.85 |
| 13 | 0.89 | 12.51 | 3.93 | 0.93 | 0.90 |
| 14 | 0.83 | 14.51 | 5.09 | 1.45 | 0.84 |
| 15 | 0.77 | 21.05 | 7.84 | 2.42 | 0.75 |
| 16 | 0.88 | 6.00 | 3.46 | 0.80 | 0.91 |
| 17 | 0.89 | 16.01 | 4.67 | 1.08 | 0.85 |
| 18 | 0.78 | 20.41 | 7.02 | 1.99 | 0.85 |
| 19 | 0.83 | 13.28 | 6.84 | 1.59 | 0.86 |
| 20 | 0.84 | 15.28 | 5.04 | 1.37 | 0.84 |

Table A3-3. Performance Metrics for the HR CTV of all 20 test patients for the 3DFR configuration.

| Test Pt | DSC | HD (mm) | HD95 (mm) | MSD (mm) | Precision |
| --- | --- | --- | --- | --- | --- |
| 1 | 0.82 | 10.00 | 7.87 | 2.71 | 0.94 |
| 2 | 0.78 | 11.75 | 9.26 | 2.86 | 0.91 |
| 3 | 0.81 | 8.12 | 5.02 | 1.78 | 0.92 |
| 4 | 0.89 | 4.05 | 2.18 | 1.02 | 0.98 |
| 5 | 0.80 | 6.68 | 6.00 | 2.68 | 0.95 |
| 6 | 0.84 | 5.36 | 3.76 | 1.46 | 0.96 |
| 7 | 0.87 | 4.56 | 3.48 | 1.42 | 0.97 |
| 8 | 0.68 | 12.04 | 10.41 | 3.97 | 0.93 |
| 9 | 0.81 | 11.29 | 6.85 | 2.36 | 0.94 |
| 10 | 0.75 | 8.24 | 8.00 | 3.22 | 0.91 |
| 11 | 0.76 | 9.09 | 6.16 | 2.91 | 0.94 |
| 12 | 0.75 | 12.35 | 9.79 | 3.44 | 0.87 |
| 13 | 0.83 | 7.67 | 4.25 | 2.25 | 0.96 |
| 14 | 0.85 | 8.24 | 6.16 | 1.61 | 0.91 |
| 15 | 0.78 | 11.12 | 6.25 | 2.22 | 0.93 |
| 16 | 0.87 | 9.42 | 4.19 | 1.34 | 0.96 |
| 17 | 0.86 | 6.31 | 5.22 | 1.62 | 0.94 |
| 18 | 0.74 | 11.83 | 5.59 | 2.40 | 0.93 |
| 19 | 0.85 | 7.42 | 6.00 | 2.01 | 0.95 |
| 20 | 0.81 | 8.29 | 6.80 | 2.00 | 0.91 |

**A4: Example of Major revisions**
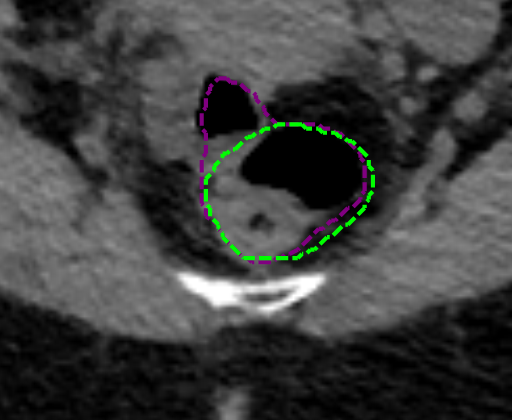

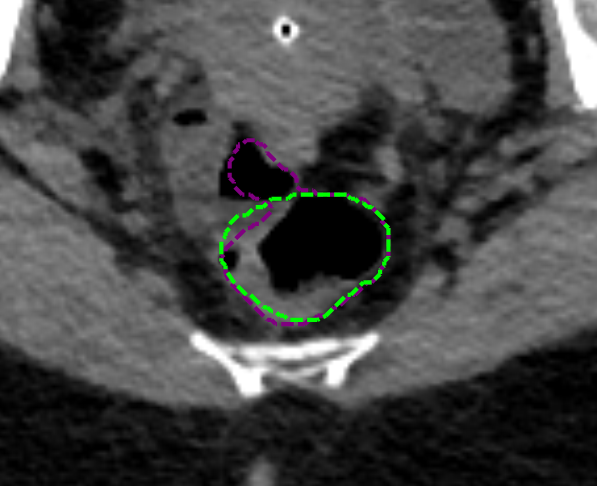


Figure A4-1. The single test patient that required major revisions on the rectum contour, where green = ground truth and purple = predicted.

**A5: nnU-net Rule-based parameters**

Table A5-1. Network configurations generated by nnU-Net.

|  | 2D | 3DFull | 3D Low-Res |
| --- | --- | --- | --- |
| Target spacing (mm): | NA x 1.00 x 1.00 | 1.00 x 1.00 x 1.00 | 1.35 x 1.35 x 1.35 |
| Median image shape at target spacing: | NA x 512 x 512 | 90 x 512 x 512 | 67 x 380 x 380 |
| Patch size: | 512 x 512 | 40 x 256 x 240 | 40 x 256 x 240 |
| Batch size: | 66 | 2 | 2 |
| Downsampling strides: | [[2, 2], [2, 2], [2, 2], [2, 2], [2, 2]] | [[2, 2, 2], [2, 2, 2], [2, 2, 2], [1,2, 2]] | [[2, 2, 2], [2, 2, 2], [2, 2, 2], [1,2, 2]] |
| Convolution kernel sizes: | [[3, 3], [3, 3], [3, 3], [3, 3], [3, 3], [3, 3]] | [[3, 3, 3], [3, 3, 3], [3, 3, 3], [3, 3, 3], [3, 3, 3]] | [[3, 3, 3], [3, 3, 3], [3, 3, 3], [3, 3, 3], [3, 3, 3]] |
